# Cultural adaptation of Kiswahili version of PedsQL 4.0 Generic Core Scales for Kenyan infants up to 24 months of age

**DOI:** 10.1186/s41687-026-01169-z

**Published:** 2026-08-06

**Authors:** Kaytlin Eastes, Eren Oyungu, Ananda Roselyne Ombitsa, Emily Abuonji, Beatrice Kaniaru, Carolyne Jerop, Cleophas Cherop, Melissa R. Thomas, Megan S. McHenry

**Affiliations:** 1 Indiana University Center for Global Health, Indianapolis, USA; 2 Department of Medical Physiology, Moi University, Eldoret, Kenya; 3 Department of Pediatrics, Indiana University School of Medicine, Indianapolis, USA; 4 AMPATH, Eldoret, Kenya

## Abstract

**Background:**

Quality of life measures are important for understanding child well-being, particularly in low- and middle-income countries where linguistic and cultural differences can limit the use of standardized tools. Although the PedsQL 4.0 Generic Core Scales (GCS) for Infants is widely used, no Kiswahili translation exists for infants in Kenya. This study aimed to culturally adapt the PedsQL 4.0 GCS for use in western Kenya.

**Methods:**

Cultural adaptation followed Mapi Research Institute guidelines. Two bilingual translators independently completed forward translations, which were reconciled into a Kiswahili version. A third translator conducted backward translation, with discrepancies resolved by a multidisciplinary committee. Cognitive interviews were conducted with nine bilingual caregivers using the adapted 13–24 month scale to assess comprehension and cultural relevance. Feedback was used to revise wording and ensure conceptual equivalence. Identical changes were applied to the 1–12 month scale.

**Results:**

Nine caregivers (89% female; mean age 32.5 years) participated in cognitive interviews. Findings resulted in 15 revisions to the Kiswahili translation, addressing literal translations that were uncommon in local usage, cultural incongruities, such as the absence of a direct Kiswahili term for “tickle”, and grammatical structures that hindered clarity. Revised versions were reviewed and approved by the Mapi Research Institute.

**Conclusion:**

Using systematic translation and cognitive interviewing, we produced a culturally relevant Kiswahili version of the PedsQL 4.0 GCS. This tool fills an important gap in measuring infant quality of life in western Kenya and is now used in ongoing research. Additional psychometric validation is needed to assess reliability, validity, and applicability across diverse caregivers.

**Supplementary Information:**

The online version contains supplementary material available at https://doi.org/10.1186/s41687-026-01169-z.

## Background

Quality of life measurements play a crucial role in assessing the well-being of populations, particularly in low- and middle-income settings (LMICs) where socio-economic challenges, health disparities, and limited access to essential services necessitate a deeper understanding of quality of life [1]. Quality of life measurements allow researchers, clinicians, and family members to determine whether an individual is not only surviving, but thriving beyond physical diagnoses and functioning. However, measuring these factors cross-culturally can be difficult due to differences in language and contextual understanding of quality of life across a variety of settings [2]. Assessment of quality of life among children adds an additional level of difficulty as it must take into account the various stages of cognitive development [3].

The PedsQL 4.0 Generic Core Scales (GCS) for Infants represent reliable, parent-proxy measures which have been developed to assess the quality of life of children across many cultural settings [4–6]. The assessment measures parents’ perceptions of their infant’s functioning across five scales: physical functioning, physical symptoms, emotional functioning, social functioning, and cognitive dimensions [5]. The PedsQL 4.0 GCS has been translated across 60 languages and demonstrated to be a valid and reliable tool across many different contexts for self-reported quality of life among healthy pediatric patients and patients living with chronic health conditions [6]. While the PedsQL 4.0 GCS has been used in older children across numerous settings, in sub-Saharan Africa, the only translation of the infant scales exists in South Africa [7], indicating a gap for young children in Kenya, where quality of life may be challenged by numerous barriers.

Cultural adaptation of standardized assessment tools is essential to attain the tool’s linguistic and conceptual equivalence. As it is often infeasible or impractical to develop unique tools in every language, we must accurately translate existing measures that can be deployed across diverse populations. We also know that many phrases and experiences, like those used to assess quality of life, are unique to the culture or population for which the original instrument was developed. Therefore, it is insufficient to merely translate instruments, but we must also adapt those translations to the specific cultural context of the target population. The goal of cultural adaptation of an instrument is to create a new one which is linguistically and conceptually equivalent to the original [8]. With increasing work in Kenya on quality of life of young children [9–11], our objective was to perform cultural adaptation of the PedsQL 4.0 GCS for infants ages 1–12 months and infants ages 13–24 months into Kiswahili for use in western Kenya.

## Methods

Cultural adaptation efforts took place in July and August 2021. The Mapi Research Institute granted use of the PedsQL 4.0 GCS for Infants 1–12 months and 13–24 months and permission for adaptation of the tool. We followed the Institute’s guidelines for cultural adaptation of the scales [8]. Each tool covered domains involving physical, emotional, social, and performance actions. Items within each domain varied slightly based, to ensure appropriateness for that age range of child.

First, two translators fluent in both English and Kiswahili each independently translated the scales using forward translation. The two Kiswahili translations were then compared against one another, and translators discussed the discrepancies and came to agreement in order to create a unified Kiswahili translation. A third translator then used backward translation to translate the scales from Kiswahili to English (US) and compare with the source material. Any discrepancies were discussed with the original translators, and edits were made to address the comments and create a new Kiswahili translation.

We conducted cognitive interviews to test comprehension of the translation and identify any additional cultural adaptation required to improve understanding. Inclusion criteria for participants of cognitive interviews were: over 18 years of age, caregivers of children under the age of ten years, and fluent in both English and Kiswahili. Written consent was obtained. Nine participants were recruited from a well-baby clinic to review the newly translated Kiswahili version of the PedsQL 4.0 Generic Core Scales for infants 13–24 months. Due to the similarity of the questions on the two infant scales, cognitive interviews were conducted only for the longer 13–24 months questionnaire. Identical translations were used for the PedsQL 4.0 GCS for infants 1–12 months.

Participants were asked to read each item on the questionnaire in Kiswahili, including all instructions, questions, and answer choices, and then state what they thought the questions were asking in English to the interviewer. If there was discrepancy between the participant’s understanding of the question and the intended question, participants were asked how they would change the wording in the interviews. These suggestions were considered and adopted by a multidisciplinary committee consisting of a bilingual psychologist, bilingual clinical officer (a mid-level clinical provider in Kenya), English-speaking pediatrician, and English-speaking public health professional.

## Results

An item-by-item comparison of the dimensions, response options, and linguistic adaptations for both infant scales is provided in Additional files 1 and 2. Numerous minor changes and considerations were discussed upon comparing the back translations to the original English translations. Nearly all of the differences noted were grounded in specificity of the wording, particularly for context-specific concepts lacking direct cultural and linguistic equivalents, which required the translating team to balance physical actions (e.g., ‘to tickle’) with their intended behavioral or emotional concepts (e.g., ‘being made to laugh’). Decisions were made based off of consensus among the bilingual translating team.

Nine participants (89% female; mean age = 32.5; mean years of schooling = 13.25; occupations included: four community health workers, two office workers, two self-employed, and one who did not disclose) participated in cognitive interviews. Following review of the interviews and findings by the multidisciplinary committee, fifteen additional changes were made to the Kiswahili text (Table 1), resulting in the final adapted versions of the PedsQL 4.0 GCS for infants 1–12 months and 13–24 months.

**Table 1:** Instances of potential mistranslation and misunderstanding in the newly adapted Kiswahili tool following cognitive interviews

Original English	Original Kiswahili Version	Final Kiswahili Version	Justification
0 - if it is never a problem	0 - ikiwa sio shida kamwe	0 - ikiwa **hajawahi kuwa** na shida kamwe	Different word choice to improve understanding
1 - if it is almost never a problem	1 - ikiwa karibu sio shida kamwe	1 - ikiwa **nadra** kamwe	Different word choice to improve understanding
Never	Hajawahi kamwe	Hajawahi **kuwa**	Grammatic change to improve understanding
Difficulty participating in active play	Ugumu kushiriki katika mchezo wa kutokaa	Ugumu kushiriki katika mchezo wa **kuchangamka**	Different word choice to more accurately communicate “active play”
Being lethargic	Kutokuwa machachari	Kuwa **mchovu**	Different word choice to more accurately communicate “lethargic”
Resting a lot	Kupumzika sana	Kupumzika **zaidi**	Different word choice to more accurately communicate “rest”
Feeling too tired to play	Kuhisi uchovu sana kuweza kucheza	Kuhisi uchovu sana **kutoweza** kucheza^**^	Grammatic change to improve understanding
Difficulty being soothed when picked up or held	Ugumu kutulizwa **wakati** anapochukuliwa au kushikwa	Ugumu kutulizwa anapochukuliwa au kushikwa	Different word choice to more accurately communicate “soothed”
Not laughing when tickled	Kutocheka anapo bembelezwa	Kutocheka **anapochekeshwa**	Different word choice to communicate a culturally relevant and functionally similar concept to “tickled”
Cognitive functioning (problems with…)	Utendaji wa utabuzi (shida na…)	**Utambuzi wa kufikiria** (shida na…)	Different word choice to more accurately communicate “Cognitive Functioning”
Not imitating caregivers’ actions	Kutoiga matendo ya mlezi	Kutoiga/**kutofanya** matendo ya mlezi	Different word choice to improve understanding
Not imitating caregivers’ facial expressions	Kutofuata ishara za uso za mlezi	Kutoiga/**kutofanya** ishara za uso za mlezi	Different word choice to improve understanding
Not imitating caregivers’ sounds	Kutoiga sauti za mlezi	Kutoiga/**kutofanya** sauti za mlezi	Different word choice to improve understanding
Not imitating caregivers’ speech	Kutoiga maongezi ya mlezi	Kutoiga/**kutofanya** maongezi ya mlezi	Different word choice to improve understanding
Difficulty pointing to his/her body parts when asked	Ugumu kuonyesha **kwa** viungo vyake vya mwili anapoulizwa	Ugumu kuonyesha viungo vyake vya mwili anapoulizwa	Grammatic change to improve understanding

* Bolded text indicates wording that was removed, added, or changed in the final Kiswahili version

** Another Kiswahili version for this phrase is: “Kuhisi uchovu sana kiasi cha kutoweza kucheza”

Many instances of confusion in the cognitive interviews were regarding statements where the multidisciplinary committee used a direct translation of the source text. In this case, certain phrases or sentence structures directly from the source material are not commonly used in Kiswahili. For example, it was suggested “ikiwa **karibu sio shida** kamwe” which translates to “almost never a problem,” be changed to “ikiwa **nadra** kamwe,” which translates to “rarely ever.” This translation is less exact than the first, but is conceptually equivalent and uses a shorter, more commonly used phrase.

There were also instances where the multidisciplinary committee attempted to directly translate concepts which are not understood in a Kenyan context. For example, the phrase “Not laughing when tickled” was not well understood during cognitive interviews. Although there is a Kiswahili word meaning “to playfully touch” in areas such as under the armpit (‘kutekenya’), it is not common for Kenyan caregivers to tickle children, and therefore there is no Kiswahili word which directly means “to tickle.” The original translation used a Kiswahili phrase which means something close to “touch” or “pet,” but this created confusion about the meaning of the question during cognitive interviews. Because the intent of this question is to assess social functioning, the multidisciplinary committee determined the Kiswahili translation should communicate the child does not laugh when someone tries to make them laugh. Ultimately, this translation was changed to “Kutocheka **anapochekeshwa**,” which translates to “Not laughing when he is made to laugh.”

The final adapted documents were reviewed and approved by the Mapi Research Institute.

## Discussion

With careful considerations of the cultural adaptations, as well as interviews to clarify understanding, new versions of the PedsQL 4.0 GCS for infants (1–12 months and 13–24 months) were developed for Kiswahili-speaking populations in western Kenya. By following an extensive protocol for cultural adaptation, we have developed a tool to accurately evaluate early quality of life within this context. The current adaptation has been incorporated into ongoing studies assessing outcomes of children born to mothers with HIV, providing valuable insights into the children’s overall well-being beyond the clinical outcomes [12].

This tool translated into the local language and adapted to the specific cultural context will be vital in assessing the well-being of infants in western Kenya. Prior cross-cultural PedsQL validations highlight the need for site-specific adaptation to ensure both linguistic accuracy and cultural tailoring [13–15]. Thoughtful adaptation of the source text to Kiswahili revealed critical linguistic and cultural differences which were addressed to better serve the target population. Similar to the current Kiswahili version, the Chichewa version (for children aged 8–12 and 13–17 years in Malawi) required substantive rephrasing of culturally incongruent items, such as “[walk] one block”, and clarification of different terms than the Kiswahili translation, such as “taking a shower or bath”, to avoid unintended meanings in Chichewa [13]. The Malawi version was further piloted and psychometrically evaluated to demonstrate internal consistency across subscales, convergent validity, and known-groups validity in sick and well children [13]. This demonstrates the localized adaptation required in each implementation site— such as in western Kenya— to ensure interpretation and relevance.

The distinction between linguistic and conceptual equivalence is particularly important in parent-proxy measures, such as the PedsQL 4.0 GCS, where caregivers must interpret abstract concepts regarding functioning and well-being. The cognitive interviews also highlighted the importance of considering who serves as the proxy respondent, as our sample leaned toward more formally educated and employed caregivers. Therefore, additional evaluation in other Kiswahili-speaking populations will allow for greater generalizability. While site-specific adaptation may reduce direct comparability with other language versions, the adapted version is strengthened by ensuring that respondents interpret questions as intended. Beyond traditional clinical outcomes, such measures can capture dimensions of functioning and caregiver perceptions that are increasingly recognized as important indicators of child health.

This adaptation also underscores the need to interpret infant quality of life items in relation to culturally patterned child experiences, rather than assuming that all developmental behaviors or caregiver–child interactions carry the same meaning across settings. For example, the item “Not laughing when tickled” required adaptation not simply because of a lexical gap, but because tickling itself may not represent a common or idealized form of caregiver–infant play in this Kenyan context; the final wording therefore shifted the item toward the broader social-emotional construct of whether the child laughs when someone attempts to make them laugh. This distinction is important because cross-cultural developmental research has shown that common assumptions in behavioral science often derive from high-income and industrialized populations and may not generalize across cultural contexts [14]. Thus, culturally adapting pediatric quality of life measures requires attention not only to linguistic equivalence, but also to whether the underlying experience being queried is developmentally and culturally salient for the population being assessed.

Some limitations of this study should be noted. First, while our cognitive interview sample size is comparable to that of other cultural adaptations of the PedsQL scales [13, 15, 16], our sample included educated participants, nearly all of whom were employed, so current translation may be less generalizable for those with less education. Second, due to time and resource constraints, no pilot testing or validation was done on the newly developed Kiswahili PedsQL 4.0 GCS Infant Scales. More work will be done in this area in the future; until then, the current adapted version should be used with caution. Additionally, we must take into account differences in child rearing practices across cultures and how those differences may influence the measurement of quality of life using this tool. In Kenya, child rearing responsibilities are often shared among family members, particularly older siblings, and the broader community [17, 18]. Therefore, additional testing across caregivers may be considered to determine if the PedsQL 4.0 GCS, which are administered as parent-proxy reports, is an appropriate tool for this cultural context.

## Conclusion

To our knowledge, this is the first documented cultural adaptation to Kiswahili of the PedsQL 4.0 GCS for Infants, filling a critical need for a culturally appropriate quality of life assessment for infants in western Kenya. Additionally, the cultural and linguistic adaptation of this tool highlights some unique differences in understanding certain cultural phenomenon within the Kenyan context, which should be considered in future adaptation of similar tools. With awareness of the lack of psychometric evaluation, the resulting adaptation of the PedsQL 4.0 GCS for Infants is now being exploratorily used in multiple ongoing studies within this population, providing valuable preliminary insights about infants’ well-being.

## Supplementary Information

Below is the link to the electronic supplementary material.

Supplementary Material 1: English back translation and changes to PedsQL 4.0 GCS (1-12 months)

Supplementary Material 2: English back translation and changes to PedsQL 4.0 GCS (13-24 months)

## Data Availability

All data and materials resulting from this study, including the translated measures, may be available through the MAPI Research Institute.

## References

[1] Paltzer J, Barker E, Witt WP (2013) Measuring the health-related quality of life (HRQoL) of young children in resource-limited settings: a review of existing measures. Qual Life Res 22:1177–1187 10.1007/s11136-012-0260-1 22983780

[2] Kagawa-Singer M, Padilla GV, Ashing-Giwa K (2010) Health-Related Quality of Life and Culture. Semin Oncol Nurs 26(1):59–67. 10.1016/j.soncn.2009.11.008 20152579

[3] Varni JW, Limbers CA (2009) The pediatric quality of life inventory: measuring pediatric health-related quality of life from the perspective of children and their parents. Pediatr Clin North Am 56(4):843–863 10.1016/j.pcl.2009.05.016 19660631

[4] Varni JW, Seid M, Rode CA (1999) The PedsQL™: Measurement Model for the Pediatric Quality of Life Inventory. Med Care 37(2):126–139 10.1097/00005650-199902000-00003 10024117

[5] Varni JW, Limbers CA, Neighbors K, Schulz K, Lieu JEC, Heffer RW, Tuzinkiewicz K, Mangione-Smith R, Zimmerman JJ, Alonso EM (2011) The PedsQL™ Infant Scales: feasibility, internal consistency reliability, and validity in healthy and ill infants. Qual Life Res 20(1):45–55. 10.1007/s11136-010-9730-5 20730626

[6] Varni JW, Burwinkle TM, Seid M (2005) The PedsQL™ as a pediatric patient-reported outcome: reliability and validity of the PedsQL™ Measurement Model in 25,000 children. Expert Rev PharmacoEcon Outcomes Res 5(6):705–719. 10.1586/14737167.5.6.705 19807613

[7] Ngwira LG, Khan K, Maheswaran H, Sande L, Nyondo-Mipando L, Smith SC, Petrou S, Niessen L (2021) Measures Applied and Validated for Use in Childhood and Adolescent Populations in Sub-Saharan Africa. Value Health Reg Issues 25:37–47. 10.1016/j.vhri.2020.11.009. A Systematic Literature Review of Preference-Based Health-Related Quality-of-Life 33765659

[8] Mapi Research Institute (2002) Linguistic Validation of the PedsQL - A quality of life questionnaire. Mapi Research Institute, Lyon, France

[9] Okech T, Thavorncharoensap M, Chaikledkaew U, Omondi GP, Nyokabi P, Thumbi SM, Thakkinstian A (2025) Determining health-related quality of life among children with malaria in Kenya: does the choice of EQ-5D value set matter? Health Qual Life Outcomes 23(1):114. 10.1186/s12955-025-02445-0 PMC12581564 41185031

[10] Spinhoven P, Zulaika G, Nyothach E, van Eijk AM, Obor D, Fwaya E, Mason L, Wang D, Kwaro D, Phillips-Howard PA (2022) Quality of life and well-being problems in secondary schoolgirls in Kenya: Prevalence, associated characteristics, and course predictors. PLOS global public health 2(12):e0001338. 10.1371/journal.pgph.0001338 PMC10022324 36962912

[11] Elson L, Otieno B, Matharu AK, Riithi N, Chongwo EJ, Mutebi F, Feldmeier H, Krücken J, Fillinger U, Abubakar A (2025) Assessing the impact of tungiasis on children’s quality of life in Kenya. PLoS Negl Trop Dis 19(9):e0012606. 10.1371/journal.pntd.0012606 PMC12431661 40920811

[12] Oyungu E, El Kebbi O, Vreeman R, Nyandiko W, Monahan PO, Tu W, Khaitan A, Desta Z, Slogrove AL, Humphrey JM, Were E, Patel RC, Carlucci JG, Wools-Kaloustian K, McHenry MS (2022) Predicting neurodevelopmental risk in children born to mothers living with HIV in Kenya: protocol for a prospective cohort study (Tabiri Study). BMJ Open 12(4). 10.1136/bmjopen-2022-061051 PMC8981283 35379648

[13] Ngwira LG, Maheswaran H, Petrou S, Niessen LW, Smith SC (2024) Cross-cultural adaptation and psychometric validation of the Chichewa (Malawi) PedsQL™ 4.0 Generic Core Scales child self-report and PedsQL™ 4.0 GCS teen self-report. J Patient-Reported Outcomes 8(1). 10.1186/s41687-024-00761-5 PMC11387560 39254899

[14] Henrich J, Heine SJ, Norenzayan A (2010) The weirdest people in the world? Behav Brain Sci 33(2–3):61–83. 10.1017/S0140525X0999152X 20550733

[15] Sato I, Soejima T, Ikeda M, Kobayashi K, Setoyama A, Kamibeppu K (2022) Reliability and validity of the Japanese version of the Pediatric Quality of Life Inventory Infant Scales. J Patient-Reported Outcomes 6(1). 10.1186/s41687-022-00416-3 PMC8800985 35092532

[16] Olival MG, Vilela TS, Hankins JS, Bendo CB, Lobo CL, Maioli MCP, Bandeira FM, Werneck GL, Soares AR (2024) Translation, transcultural adaptation, and validation of PedsQL 3.0 Sickle Cell Disease Module into Brazilian Portuguese. Pediatr Blood Cancer 71(4). 10.1002/pbc.30862 38287205

[17] Whaley SE, Sigman M, Beckwith L, Cohen SE, Espinosa MP (2002) Infant-Caregiver Interaction in Kenya and the United States:The Importance of Multiple Caregivers and Adequate Comparison Samples. J Cross-Cult Psychol 33(3):236–247. 10.1177/0022022102033003002

[18] Jelliffe DB, Bennett FJ (1975) Aspects of child rearing in Africa. J Afr Stud 2(3):363 10.1093/tropej/18.1.25 4261852

