# Supplementary material for: Cultural adaptation of Kiswahili version of PedsQL 4.0 Generic Core Scales for Kenyan infants up to 24 months of age
Source: J Patient Rep Outcomes. 2026 Aug 6;10:201. doi: 10.1186/s41687-026-01169-z (PMC13737067; doi:10.1186/s41687-026-01169-z)
Supplement: Supplementary file 1 [file 41687_2026_1169_MOESM1_ESM.doc]

ID#:

Date:

PedsQL^™^

Children's Quality of Life Count

Infant Scales

PARENT REPORT FOR CHILDREN (ages 13-24 months)

**DETAILS**

On the next page is a list of things that can be a problem for your child.

Please tell us how much of a problem each has been a problem to your child in ONE month that has passed, with repetition:

**0 if not a problem at all**

**1 if almost never a problem**

**2 if sometimes it is a problem**

**3 if it is often a problem**

**4 if almost always a problem**

There are no right or wrong answers.

If you do not understand the question, please ask for help.

In the last 1 month, what problem has your child had with?

| **PHYSICAL WORK (trouble with ...)** | He has never been | Rarely | At other times | Many times | Almost always |
| --- | --- | --- | --- | --- | --- |
| 1. Low energy level | 0 | 1 | 2 | 3 | 4 |
| 2. Difficulty participating in the playful game | 0 | 1 | 2 | 3 | 4 |
| 3. Having injuries or pain | 0 | 1 | 2 | 3 | 4 |
| 4. Feeling tired | 0 | 1 | 2 | 3 | 4 |
| 5. Not being active | 0 | 1 | 2 | 3 | 4 |
| 6. Very relaxed | 0 | 1 | 2 | 3 | 4 |
| 7. Being very tired to play | 0 | 1 | 2 | 3 | 4 |
| 8. Difficulty walking | 0 | 1 | 2 | 3 | 4 |
| 9. Difficulty running short distances without falling | 0 | 1 | 2 | 3 | 4 |

| PHYSICAL SYMPTOMS (Trouble with…) | He has never been | Rarely | At other times | Many times | Almost always |
| --- | --- | --- | --- | --- | --- |
| 1. Having gas | 0 | 1 | 2 | 3 | 4 |
| 2. Spitting after eating | 0 | 1 | 2 | 3 | 4 |
| 3. Difficulty breathing | 0 | 1 | 2 | 3 | 4 |
| 4. Being sick in his stomach | 0 | 1 | 2 | 3 | 4 |
| 5. Difficulty swallowing | 0 | 1 | 2 | 3 | 4 |
| 6. Constipation | 0 | 1 | 2 | 3 | 4 |
| 7. Having a rash | 0 | 1 | 2 | 3 | 4 |
| 8. Having diarrhea | 0 | 1 | 2 | 3 | 4 |
| 9. Breathing loudly | 0 | 1 | 2 | 3 | 4 |
| 10.Vomiting | 0 | 1 | 2 | 3 | 4 |

| EMOTIONAL ACTIVITY (trouble with…) | He has never been | Rarely | At other times | Many times | Almost always |
| --- | --- | --- | --- | --- | --- |
| 1. Feeling worried or scared | 0 | 1 | 2 | 3 | 4 |
| 2. Feeling angry | 0 | 1 | 2 | 3 | 4 |
| 3. Weeping or complaining about being left alone | 0 | 1 | 2 | 3 | 4 |
| 4. Difficulty calming oneself down when angry | 0 | 1 | 2 | 3 | 4 |
| 5. Difficulty sleeping | 0 | 1 | 2 | 3 | 4 |
| 6. Crying or arguing while being hugged | 0 | 1 | 2 | 3 | 4 |
| 7. Feeling sad | 0 | 1 | 2 | 3 | 4 |
| 8. Difficulty being calmed when picked or held | 0 | 1 | 2 | 3 | 4 |
| 9. Difficulty sleeping especially at night | 0 | 1 | 2 | 3 | 4 |
| 10. Too much crying | 0 | 1 | 2 | 3 | 4 |
| 11. Feeling angry quickly | 0 | 1 | 2 | 3 | 4 |
| 12. Difficulty getting to sleep during the day | 0 | 1 | 2 | 3 | 4 |

*In the last 1 month, what problem did your child have with…*

| SOCIAL PERFORMANCE (Trouble with…) | He has never been | Rarely | At other times | Many times | Almost always |
| --- | --- | --- | --- | --- | --- |
| 1. Not smiling at others | 0 | 1 | 2 | 3 | 4 |
| 2. Don't laugh when they are made to laugh | 0 | 1 | 2 | 3 | 4 |
| 3. No Eye contact with the caregiver | 0 | 1 | 2 | 3 | 4 |
| 4. Not laughing when he is hugged | 0 | 1 | 2 | 3 | 4 |
| 5. Not worrying when around other children | 0 | 1 | 2 | 3 | 4 |

| **PERFORMANCE ACTION (difficulty with…)** | He has never been | Rarely | At other times | Many times | Almost always |
| --- | --- | --- | --- | --- | --- |
| 1. Not imitating the actions of a caregiver | 0 | 1 | 2 | 3 | 4 |
| 2. Not following the facial expressions of the caregiver | 0 | 1 | 2 | 3 | 4 |
| 3. Not imitating the voices of the caregiver | 0 | 1 | 2 | 3 | 4 |
| 4. Loss of focus on things | 0 | 1 | 2 | 3 | 4 |
| 5. Does not imitate the caregiver's conversation | 0 | 1 | 2 | 3 | 4 |
| 6. Difficulty showing his body parts when asked | 0 | 1 | 2 | 3 | 4 |
| 7. Difficulty naming common objects | 0 | 1 | 2 | 3 | 4 |
| 8. Difficulty repeating words | 0 | 1 | 2 | 3 | 4 |
| 9. Difficulty keeping his focus on things | 0 | 1 | 2 | 3 | 4 |
