# Supplementary material for: Cultural adaptation of Kiswahili version of PedsQL 4.0 Generic Core Scales for Kenyan infants up to 24 months of age
Source: J Patient Rep Outcomes. 2026 Aug 6;10:201. doi: 10.1186/s41687-026-01169-z (PMC13737067; doi:10.1186/s41687-026-01169-z)
Supplement: Supplementary file 2 [file 41687_2026_1169_MOESM2_ESM.doc]

ID#:

Date:

PedsQL^™^

Calculation of children's quality of life

Infant scales

PARENT REPORT FOR CHILDREN (Ages 1-12 months)

#	DETAILS

On the next page, there is a list of things that can be a problem for your child.

Please tell us how much of a problem each one here has been a problem to your child in the past one month by circuling it.

**0 If its not a problem**

**1 If its not near a problem at all**

**2 If it is sometimes a problem**

**3 If it is often a problem**

**4 If it is almost always a problem**

There are no right or wrong answers

If you do not understand the question, please ask for help

In the last one month, how much of a problem has your child had with….

| PHYSICAL WORK (trouble with ...) | He has never been | Rarely | At other times | Many times | Almost always |
| --- | --- | --- | --- | --- | --- |
| 1. Low energy levels | 0 | 1 | 2 | 3 | 4 |
| 2. Difficulty participating in the playful games | 0 | 1 | 2 | 3 | 4 |
| 3. Having injuries or pain | 0 | 1 | 2 | 3 | 4 |
| 4. Feeling tired | 0 | 1 | 2 | 3 | 4 |
| 5. not being active | 0 | 1 | 2 | 3 | 4 |
| 6. Too much rest | 0 | 1 | 2 | 3 | 4 |

| PHYSICAL SYMPTOMS (trouble with…) | He has never been | Rarely | At other times | Many times | Almost always |
| --- | --- | --- | --- | --- | --- |
| 1. Having gas | 0 | 1 | 2 | 3 | 4 |
| 2. Spitting after eating | 0 | 1 | 2 | 3 | 4 |
| 3. Difficulty breathing | 0 | 1 | 2 | 3 | 4 |
| 4. Being sick on his stomach | 0 | 1 | 2 | 3 | 4 |
| 5. Difficulty swallowing | 0 | 1 | 2 | 3 | 4 |
| 6. Constipation | 0 | 1 | 2 | 3 | 4 |
| 7. Having a rash | 0 | 1 | 2 | 3 | 4 |
| 8. Having diarrhea | 0 | 1 | 2 | 3 | 4 |
| 9. breathing loudly | 0 | 1 | 2 | 3 | 4 |
| 10. Vomiting | 0 | 1 | 2 | 3 | 4 |

| EMOTIONAL ACTIVITY (trouble with…) | He has never been | Rarely | At Other times | Many times | Almost always |
| --- | --- | --- | --- | --- | --- |
| 1. Feeling scared or fearing | 0 | 1 | 2 | 3 | 4 |
| 2. Feeling angry | 0 | 1 | 2 | 3 | 4 |
| 3. Crying or arguing when left alone | 0 | 1 | 2 | 3 | 4 |
| 4. Difficulty calming yourself down when you are angry | 0 | 1 | 2 | 3 | 4 |
| 5. Difficulty sleeping | 0 | 1 | 2 | 3 | 4 |
| 6. Crying or arguing while being hugged | 0 | 1 | 2 | 3 | 4 |
| 7. Feeling sad | 0 | 1 | 2 | 3 | 4 |
| 8. Difficulty being calmed down when picked or held | 0 | 1 | 2 | 3 | 4 |
| 9. Difficulty sleeping especially at night | 0 | 1 | 2 | 3 | 4 |
| 10. Too much crying | 0 | 1 | 2 | 3 | 4 |
| 11. Feeling angry quickly | 0 | 1 | 2 | 3 | 4 |
| 12. Difficulty getting to sleep during the day | 0 | 1 | 2 | 3 | 4 |

IN THE LAST MONTH, HOW MUCH OF A PROBLEM HAS YOUR CHILD HAD WITH ……………

| SOCIAL PERFORMANCE (PROBLEMS WITH…) | He has never been | Rarely | At other times | Many times | Almost always |
| --- | --- | --- | --- | --- | --- |
| 1. Not smiling at others | 0 | 1 | 2 | 3 | 4 |
| 2. Not Laughing when he is made to laugh | 0 | 1 | 2 | 3 | 4 |
| 3. No Eye contact with caregiver | 0 | 1 | 2 | 3 | 4 |
| 4. Not laughing when hugged | 0 | 1 | 2 | 3 | 4 |

| **IDENTIFICATION WORK (PROBLEMS WITH…)** | He has never been | Rarely | At other times | Many times | Almost always |
| --- | --- | --- | --- | --- | --- |
| 1. Not imitating the actions of a caregiver | 0 | 1 | 2 | 3 | 4 |
| 2. Not following the facial expressions of the caregiver | 0 | 1 | 2 | 3 | 4 |
| 3. Not imitating the voices of the caregiver | 0 | 1 | 2 | 3 | 4 |
| 4. Loss of focus on things | 0 | 1 | 2 | 3 | 4 |
